# Using social robots for language learning: are we there yet?

**DOI:** 10.1515/jccall-2023-0013

**Published:** 2023-07-10

**Authors:** Guanyu Huang, Roger K. Moore

**Affiliations:** University of Sheffield, Sheffield, UK

**Keywords:** Chinese as a foreign language (CFL), educational robots, human-robot interaction (HRI), robot-assisted language learning (RALL), technology-based teaching

## Abstract

Along with the development of speech and language technologies and growing market interest, social robots have attracted more academic and commercial attention in recent decades. Their multimodal embodiment offers a broad range of possibilities, which have gained importance in the education sector. It has also led to a new technology-based field of language education: robot-assisted language learning (RALL). RALL has developed rapidly in second language learning, especially driven by the need to compensate for the shortage of first-language tutors. There are many implementation cases and studies of social robots, from early government-led attempts in Japan and South Korea to increasing research interests in Europe and worldwide. Compared with RALL used for English as a foreign language (EFL), however, there are fewer studies on applying RALL for teaching Chinese as a foreign language (CFL). One potential reason is that RALL is not well-known in the CFL field. This scope review paper attempts to fill this gap by addressing the balance between classroom implementation and research frontiers of social robots. The review first introduces the technical tool used in RALL, namely the social robot, at a high level. It then presents a historical overview of the real-life implementation of social robots in language classrooms in East Asia and Europe. It then provides a summary of the evaluation of RALL from the perspectives of L2 learners, teachers and technology developers. The overall goal of this paper is to gain insights into RALL’s potential and challenges and identify a rich set of open research questions for applying RALL to CFL. It is hoped that the review may inform interdisciplinary analysis and practice for scientific research and front-line teaching in future.

## 1 Introduction

With the development of technology, the means of assisting language teaching and learning have become more and more diverse. The emergence and popularity of computers have opened the field of Computer Assisted Language Learning (CALL) since the 1960s (Allen, 1972). Going beyond the capabilities of storing and playing learning materials like tapes and CDs, CALL has provided an interactive approach to language education across all learning stages. In recent years, such an approach has been brought further by the rapid development of robotics, especially speech-enabled social robots. A new area has emerged on the map of language education, which is Robot-Assisted Language Learning (RALL).

RALL is defined as using robots to teach people native or non-native language skills, including sign languages (Randall, 2019). Within the broad scope of human-robot interaction (HRI), RALL is a subfield of robot-assisted learning (RAL or R-learning). One of the first publications of RALL could be dated back to 2004 by computer scientists Kanda et al. (Kanda et al., 2004). It was about an 18-day field trial held at a Japanese elementary school with the main goal of studying how to ‘create partnerships in a robot’. More than a decade on, RALL has attracted the attention of more researchers. The questions identified in Kanda’s research back then, such as a rapidly dissipating novelty in HRI, still await better solutions.

Looking at the development of CALL from birth to maturity, it is clear that the development of technology-based language teaching relies on two pillars: the technology itself and the way in which the technology is applied based on pedagogy. RALL is still in its early stage of development. People are still exploring whether it is worth using and how to use it. Thus, the purpose of this review article is twofold. The first aim is to outline an overview of the RALL field. The second aim is to identify possible research directions for relevant technology developers and teachers.

The paper is organised around the proposed aims as follows. It starts with an introduction of robots used in the RALL, including their types, functions and their usage in existing studies. It then provides a historical review of how robots have been used in real-life classrooms, especially in East Asia and Europe. It is followed by signposting the aspects of RALL evaluation regarding language skills and teaching strategies, as well as the challenges for robot development. It concludes with implementation guidelines for front-line language teachers who would like to use social robots in their classrooms. It also summarises a set of open research questions for robot developers for future RALL research.

## 2 Technological background: social robots for language learning

### 2.1 From industrial robots to social robots

Robots are machinery agents which can carry out a series of actions automatically, based on pre-set programmes. They are equipped with hardware and software to collect information, process signals, and convert electrical signals into physical movement (Bartneck et al., 2020, pp. 18–37). In comparison with robots used in the industrial domain, which mainly focus on completing physical tasks, social robots are expected to communicate and interact with people on an emotional level (Darling, 2016). With the rapid development of speech and language technologies in recent decades, social robots have gained the ability to perceive, process and produce speech, such as automatic speech recognition (ASR) or text-to-speech (TTS), natural language processing (NLP) and text-to-speech (TTS). In other words, robots can interact with human users via speech.

This speech-enabled capability has fostered robots’ transition from the industrial domain to social domains, such as service industries, healthcare, entertainment and education (Bartneck et al., 2020, p. 163). In this last regard, robots, as pedagogical tools, are not only popular for science, technology, engineering and maths (STEM) education but also show great promise in social interaction with increasing cognitive and affective outcomes (Belpaeme et al., 2018). The popularity of using social robots in educational environments has been increasing over the years. Analysts expect the robotics education market to reach a market value of $2.6 billion by 2026 (MarketsandMarkets, 2021). Language learning is one of the three major application areas for social robots (Mubin et al., 2013).

### 2.2 Educational social robots

#### 2.2.1 Features, advantages and myths

In comparison with traditional digital learning tools used for language learning and teaching, a distinct advantage that social robots have is their physical embodiment. Social robots’ embodiment tends to be multimodal. It combines multiple sensors, actuation and locomotion. Thus, it can offer a wider range of interactive possibilities in language classrooms than other forms of technology (e.g., tablets, computers and smartphones). For example, it can interact with the learning environment and learners physically. As shown in (de Wit et al., 2018), the robot’s use of gestures positively affected students’ long-term memorisation of words in the second language (L2).

As early as 1986, Harwin, Ginige and Jackson proposed using robots for physical interaction in early education (Harwin et al., 1986). In addition, studies have shown that the presence of social robots can (1) help students achieve better task performance compared to virtual agents or robots displayed on screens (Leyzberg et al., 2012; Li, 2015) and (2) increase people’s evaluation of robots and their interactions with robots by making robots appear more appealing, perceptive and enjoyable (Jung & Lee, 2004; Wainer et al., 2007). This advantage is likely attributable to many factors. One major reason could be robots’ positive effects on learning motivation, which could be very rewarding for second language acquisition, according to Krashen and Terrel’s Affective Filter Hypothesis (Krashen, 2009). Such a positive connection between robots’ embodiment and motivation has been found in many types of robot-assisted learning, yet generally not found in other types of technology (Van den Berghe et al., 2019).

However, social robots’ ability to motivate students is not completely clear. Students’ enthusiasm could be sparked by the new technology, which would not have been sustained over long periods, as discussed in Van den Berghe et al.’s review in 2019 (Van den Berghe et al., 2019). Besides this novelty effect, social robots’ physical presence could also cause unexpectedly worse performance. In a study investigating children’s grammar learning, children performed worse when the robot looked at them (Herberg et al., 2015). It is unclear whether such counterproductive effects were caused by increased pressure (a possible explanation provided by the experimenters in Herberg et al. (2015)) or increased distraction or even fear. Apart from that, robots’ presence may also cause negative effects if they do not have the touch-input capability, as shown in a study in 2004 (Jung & Lee, 2004). The study has flagged the importance of (1) bridging users’ expectations of an embodied robot and its capability and (2) using tactile communication (not necessarily via a touch screen) to enhance robots’ social presence.

**Figure 1:**
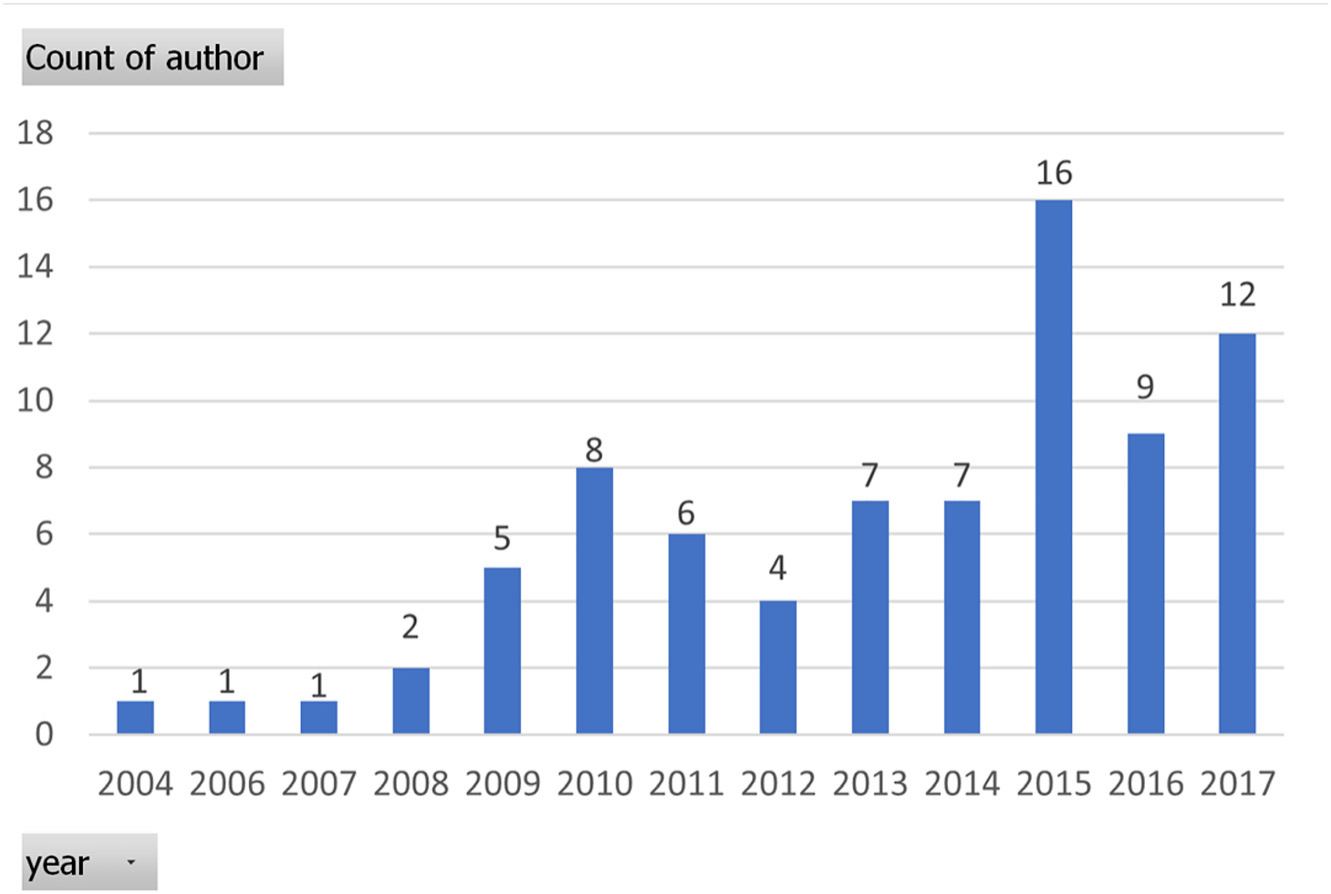
Numbers of published RALL papers from 2004 to 2017.

Added to motivating students, social robots also have many advantages, such as their capability when handling repeated tasks without fatigue. Also, they have the flexibility to be programmed to take up various roles in the classroom, such as teachers or learning companions (Aidinlou et al., 2014; Mubin et al., 2013). These potential advantages of social robots also come with many issues that need to be addressed, including technology limits, robots’ credibility and explainability, usability and social impacts on language teaching and learning. These will be addressed in the implementation section later. Based on the extensive review by Randall in 2019 (Randall, 2019), which includes 79 papers about RALL, it is clear that interest and enthusiasm about RALL have been on the rise over the past decade (shown in Figure 1). Along with the rapid growth of communication technology and robotics, it is anticipated that RALL will keep growing in the coming decades.

#### 2.2.2 Types by autonomous function and appearance

According to Randall’s review in 2019 (Randall, 2019), at least 26 different social robots were used in 79 RALL studies from 2004 to 2017. The review provides a detailed description of the characteristics of robots used in RALL, such as autonomous functions, forms, voices, social roles, verbal and non-verbal immediacy and personalization. Among these features, robots are often categorised by their autonomous functionality and appearances.

**Figure 2:**
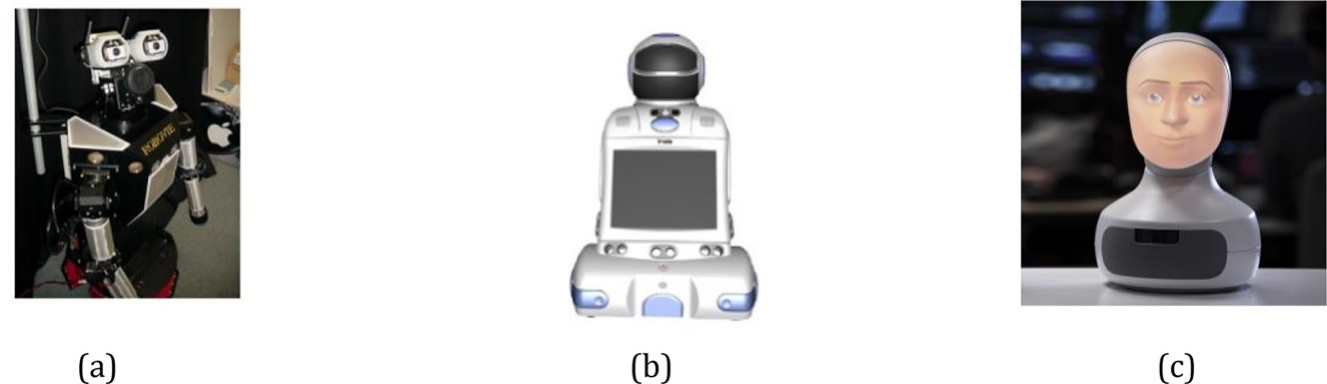
Robots used in RALL depending on their autonomous functionalities. (a) Autonomous Robot: Robovie, (b) Teleoperated Robot: iRobi, (c) Transformable Robot: Furhat.

According to the degree of automation, robots used in RALL can be: (1) fully autonomous: acting upon predefined programmes and generating contingent responses when the participants behave as expected; (2) fully teleoperated (or telepresent): being operated remotely by the people to generate more flexible real-time (re)action; (3) transformed: being between the above two levels of operations (Han, 2012). Although it is natural to test the usability of a fully autonomous robot, all three types of robots are often used in RALL because they can serve different purposes. For example, due to technical limitations of handling automatic speech recognition (ASR) of non-native speakers, as well as limited incremental dialogue systems, robots may have difficulties understanding non-native language learners’ speech, not to mention adapting their speech behaviours accordingly (e.g., speech rate, rephrase, emphasis specific parts). Thus, teleoperated or semi-teleoperated robots are more suitable for studying advanced interactions in order to identify appropriate robot properties or behaviours for further development. Some examples of these robots are provided in Figure 2 (Furhat Robotics, 2023; Ishiguro et al., 2001; Lee et al., 2006; Yun et al., 2011).

**Figure 3:**
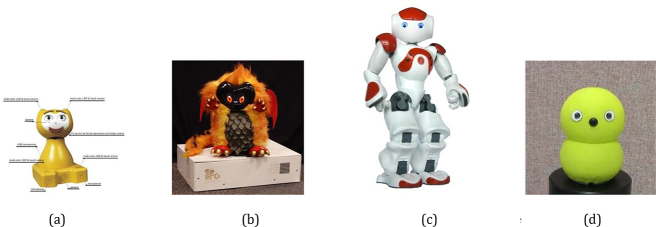
Robots used in RALL depending on their forms. (a) Zoomorphic Robot: iCat robot, (b) Carton-like Robot: DragonBot, (c) Human-like Robot: NAO, (d) Minimal Social Robot: Keepon.

**Figure 4:**
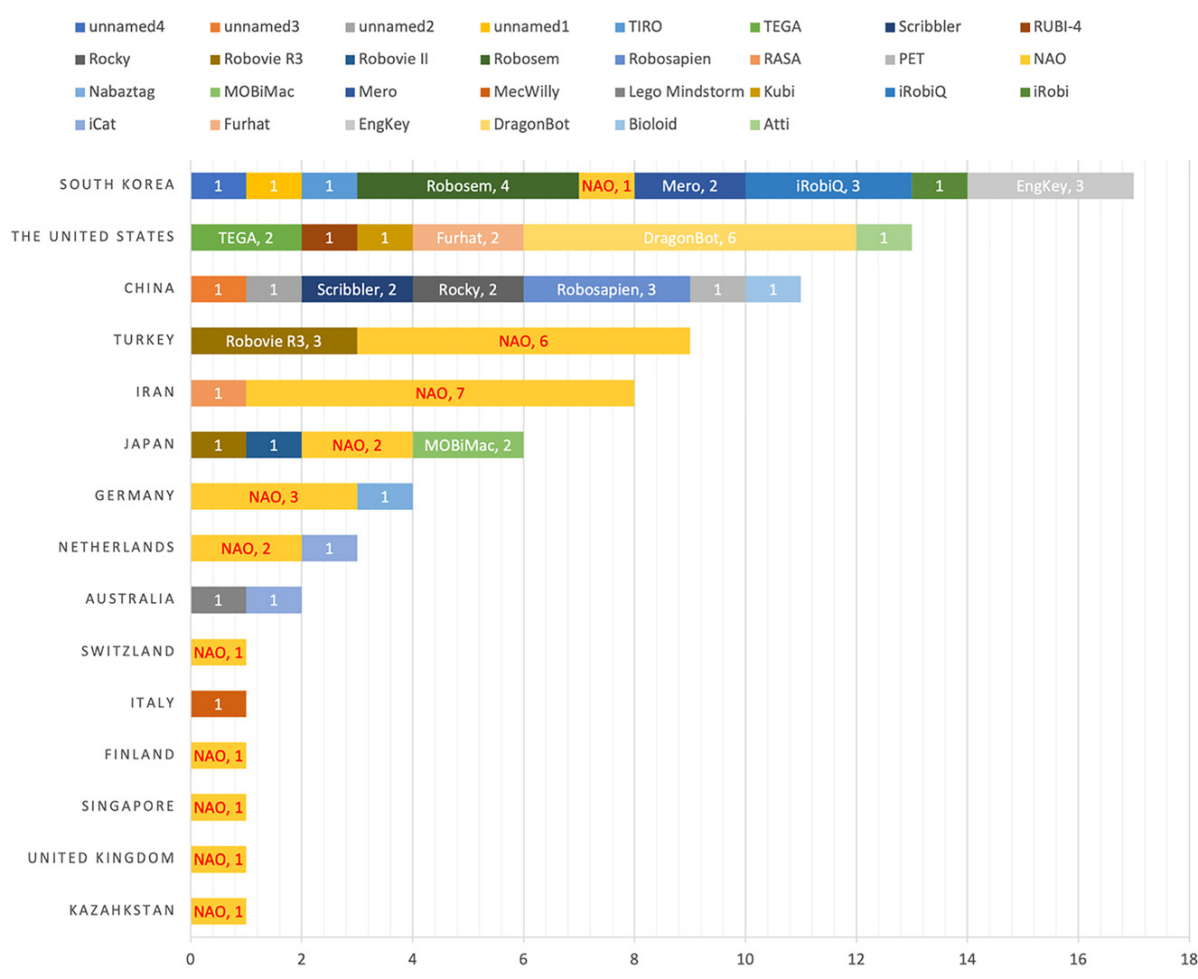
Robots used in RALL research by countries and regions (2004–2017).

Robots used in RALL can also be categorised by their forms. Robots’ forms and functions are interconnected (Bartneck et al., 2020, p. 43). In other words, the form of a robot represents how it can interact with people and also sets physical constraints. For language learning purposes, robots have facial expressions, body gestures and speech-enabled capabilities that are preferable. Hence, differentiating from companion robots that could be beneficial to have an animal-like form with limited functions (e.g., a seal-like robot companion “Paro”1Paro is an advanced interactive robot, mostly used in the eldercare domain. To provide companion service, Paro is equipped with sensors to detect “when it is being picked up or stroked” and respond by “wriggling and making seal-like noises” (Bartneck et al., 2020, pp. 170–171). http://www.parorobots.com.), robots used in RALL are often equipped with more human-like characteristics or even human-like forms. As shown in Figure 3, some RALL robots are still zoomorphic but built with more expressive functions, like iCat (van Breemen et al., 2005); some are cartoon-like, like DragonBot (MIT Media Lab, n.d.); some are more human-like, which is known as being anthropomorphic, like NAO (Gouaillier et al., 2009),2NAO is created by SoftBank Robotics. https://www.softbankrobotics.com/emea/en/nao. which is currently the most popular research platform in social robotics (Bartneck et al., 2020, p. 14) (Randall, 2019) (as shown in Figure 4). Robots like “NAO” are called humanoid robots, which resemble the human body in shape, partially or fully. A minimal social robot, “Keepon”, has also been popular in RALL studies. It is shown that the simple form is sufficient to achieve the expected interaction outcomes (Kozima et al., 2009).

This wide range of options has provided many possibilities in RALL studies, especially the research hotspot of children-robot interaction in RALL. For the robots used in 79 relevant RALL studies (2004–2017), Randall has provided a detailed table in the review (Randall, 2019, p. 7: 5). On the basis of Randall’s table, it can be seen that most areas involved in RALL have used only one or two types of robot. South Korea has used the largest variety of robots in RALL (as shown in Figure 4). One possible reason could be that the choice of a robot to use in RALL studies is often related to the availability and affordability of the robot. Nevertheless, where possible, the choice of the robot should also consider the perception of the robot by the teaching audience, including its size, appearance and cultural suitability. Perception matters because, according to affordance theory (Gibson, 1977; Matei, 2020), perception affects expectations and interaction with the robot. For those interested in robots’ characteristics, Van de Bergh et al. have summarised them in a clear table (Van den Berghe et al., 2019, p. 287). It is also worth bearing in mind that robots’ human likeness is not directly linked with their expressiveness, as illustrated in Engwall and Lopes’s review in 2022 (Engwall & Lopes, 2022). The current RALL is generally applied to younger learners. As future RALL research pays more attention to adult second language acquisition, it is hoped to see more adult-friendly language teaching robots with appropriate forms.

## 3 Implementation of social robots in language classrooms

### 3.1 RALL in East Asia

Early research and commercialisation of RALL began in countries and regions where English has been popular as a second language to acquire. In East Asia, the market for teaching English as a second language is huge, and the development of robotics research is at the forefront of the world. Thus, it is easy to imagine why Asian countries have explored and experimented with language-teaching robots earlier and to a great extent. To address the dilemma of the lack of native English teachers in their home countries, South Korea and Japan have successively experimented with robot teachers with the support of their governments.

#### 3.1.1 South Korea

According to the reports (Susannah Palk, CNN, 2010). In 2010, the Korea Institute of Science and Technology (KIST), with government funding, developed a robot teacher named “Engkey”, which has the body of a robot with its face replaced by a screen (one example of the teleoperated robot, shown in Figure 2 in Session 2.2.2). Engkey is designed for primary school classes, where it acts as a teaching assistant and interacts with the teacher and students in the classroom, for example, by doing simple pronunciation and dialogue exercises. After the pilot programme at two elementary schools in 2010, Engkey, along with other developed robots for R-Learning, has been rolled out on a wider scale. By 2014, over 1,500 robots were used for playing activities and attitude training, and over 30 English instructional robots were utilised in elementary after-school activities in South Korea (Han, 2012). In terms of effectiveness, Engkey helps to increase students’ interest and enthusiasm in learning, improve their concentration in class, and in turn, improve their English skills. However, some experts have shown concerns that young students may develop attachment disorders when using robot helpers for long periods. The government has planned to expand the use of Engkey as it is effective, relatively cost-effective and easy to maintain (Wright, 2021).

#### 3.1.2 Japan

As for Japan, the Tokyo University of Science developed the world’s first robot teacher “Saya” in 2009 after 15 years of work (The Guardian, 2009): It is a highly realistic-looking teacher who can make six basic expressions and has limited functionality, except for registering attendance and shouting, “Be quiet!”. The widespread use of robot teachers in Japanese schools is slightly later than in South Korea. According to the report (Kyodo News, 2018), since 2011, English has been a compulsory subject in Years 5 and 6 in primary school on the Japanese national curriculum. In 2016, some schools in Kyoto experimented on a small scale with robotic language teachers to supplement classroom teaching. According to another report (Phys.org, 2018), from 2020, the starting grade for English as a compulsory subject in Japanese primary schools was lowered from Years 5 to 3. To fit this change into the 2020 syllabus within the limited funds available, the robot language teachers became the first choice of the Japanese Ministry of Education. In April 2018, this Ministry decided to spend around 250 million yen (approx. 2 million US dollars) to put 500 English robots into schools in 2019. These robots were used to improve students’ speaking and writing skills by working together with apps on tablets.

One of the robots used in the campaign to popularise robot English teachers is called Musio X. As reported (Hamakawa, 2018), it is only 20 cm tall and was developed by a US company AKA. The robot has “learned” millions of bytes of conversational data from American TV shows and other English language resources. It can talk freely with students outside of regular conversation practice. Some students have reported that the robot teacher’s English pronunciation is clear and easy to understand and that they are not afraid to make mistakes in front of the robot teacher, even when they have to repeat several times. Some teachers have commented that the robot language teacher has improved their students’ confidence in conversation. Also, their students spoke English at a louder volume. In addition, real teachers can track their student’s academic performance through the robot teacher’s database, and teachers feel less burdened with grading assignments.

In 2021, AKA launched a new generation version of Musio X, which is called “Musio S”. In addition to optimisations in hardware and software, Musio S adds the artificial intelligence engine “Muse”. “Muse” can also analyse learning data, with data visualisation and customised learning suggestions, to help students master conversational skills in different scenarios and topics. AKA has reportedly partnered with Oxford University Press to provide real-time feedback on learning materials based on *Let’s Go!*, the world’s leading English education programme for children (AKA, 2021b). In addition, more than 100 educational institutions in Japan and South Korea have used Musio as a smart teaching tool for regular classes (AKA, 2021a).

Another robot used in Japanese language classes is NAO (shown in Figure 3 in Session 2.2.2), developed by SoftBank Japan, which stands 58 cm tall, can hear, see and speak, and can interact with people. Another robot, Pepper, developed by SoftBank, is 120 cm tall and can judge users’ emotions based on their expressions and voice tone. There are many examples of using NAO and Pepper in the education sector. SoftBank Communications in Singapore and Nanyang Technological University have partnered to use the two robots in preschool classes, for instance, to listen to Pepper tell stories and answer questions (Government Technology Agency of Singapore, 2016). Some schools in London are using Pepper to engage students in autonomous, motivated learning (Jane Wakefield, BBC, 2017).

For language teaching, AKA has helped SoftBank Robotics develop three functions-free chat, beginner chat and teaching aid mode – to meet the flexible needs of classroom teaching. The report states that teachers can upload classroom materials through a designated website to achieve customised goals. The uploaded material can be seamlessly integrated with Pepper, which makes it possible for students to practice what the teacher has uploaded. In addition, as students practice with Pepper, AKA’s analytics software records and processes the conversation data. Teachers can easily track each student’s progress and view learning results on the website (AKA, 2020).

Pepper was launched in 2014. Attempts have been made to try to integrate with the market through innovation. While it has shone at research and educational conventions, commercial demand has been weak, and the production of Pepper was suspended in June 2021. Production was halted partly due to Pepper’s high $1,790 price tag and $360 per month subscription fee and partly because researchers believed that Pepper’s conversations were mainly controlled remotely through humans, giving a false impression of the capabilities of real-world artificial intelligence (Jane Wakefield, BBC, 2021).

#### 3.1.3 China

Whilst South Korean and Japanese RALL has been motivated to respond to the shortage of first-language speakers in English, few pieces of research have been found regarding how RALL has been developed in the Chinese mainland. It is potentially due to the limit of the search engine, which is Google Scholar, used in this review paper. Another reason could be that RALL is not well-known in the CFL field. Nevertheless, RALL researchers have been very active in Chinese Taiwan (as shown in Figure 4). Their studies have reflected the diversity of learner groups. RALL for EFL in Chinese Taiwan has covered young learners (Yin et al., 2022) and adults, such as university freshmen (Shen et al., 2019). As for CFL, Chinese scholars tried to conduct automatic oral tests for college Chinese learners by using the speech-to-text (STT) technology on a robot (Li et al., 2021). They also used RALL to teach Chinese to Vietnamese children from transnational marriages (Weng & Chao, 2022), and to promote interpersonal communication and seek well-being for new immigrants from Southeast Asians (mainly Indonesian) (Tseng & Paseki, 2022).

There is a recent RALL study on CFL conducted in Shanghai. It shows that adult Chinese learners have a higher level of engagement with embodied robots than with virtual agents (Nomoto et al., 2022). This study is based on an empirical experiment focusing on vocabulary learning. Ten students were divided into two groups: one interacting with an embodied robot and the other interacting with virtual agents. In each group, an instructor was present, and three types of interactions were conducted: a translation mode, a quiz mode and a chat mode. It was reported that the group with the physical agent ‘had higher levels of engagement and lower levels of discouragement’ (Nomoto et al., 2022). The study has discussed shortcomings, such as the small sample size, mixed levels of students’ Chinese abilities and the restriction of STT technology in vocabulary learning, especially in a tonal language like Chinese. However, it also shows some possibilities for the RALL research, including the feasibility of building a language-learning-assisted robot and getting language teachers involved in the design process.

### 3.2 RALL in Europe

Compared to RALL in Asia, the use of robotic language teachers in Europe has a shorter history and is less widespread. One of the larger projects in the early years was *Second Language Tutoring using Social Robots* (L2TOR3http://www.l2tor.eu/.). The project was funded by the European Commission’s Horizon 2020 programme and ran from 1 January 2016 to 31 December 2018. Using NAO robots, the project studied how children aged 4–6 learn a second language, with the help of social robots, through interaction with NAO. Examples include how native speakers of Dutch, German and Turkish learn English vocabulary, how native English-speaking children learn French vocabulary and grammar, and how children who have immigrated to the Netherlands from Turkey learn Dutch vocabulary.

In the classroom, the interactive human-computer platform is usually a tablet with learning games that create learning situations for students. Students sat next to the NAO, listened to the NAO interpret the game, repeated the words with the NAO’s voice and movements, or touched the tablet to interact with the NAO. The project’s main focus was a large-scale field study of 200 Dutch children learning 34 English words in seven lessons with the help of the NAO robot. The findings show that children can acquire words through interaction with NAO but that the same results can be achieved if NAO was removed and a regular tablet was used (Vogt et al., 2019). The L2TOR project has produced a rich body of research, including five focused articles, six PhD theses and several other journal articles from 2015 to 2022.4See the publication web page: http://www.l2tor.eu/researchers-professionals/publications. These have contributed to a greater understanding of the role, impact, challenges and opportunities of social robots for children’s second language acquisition.

In the Nordic region, a study called “*Collaborative Robot-Assisted Language Learning*” (CORALL5https://www.kth.se/profile/engwall/page/corall-collaborative-robot-assisted-language-learning.) is being funded by the Swedish Research Council for 2017–2020. Its social relevance is to contribute to more effective Swedish-language immigrant education by combining collaborative learning pedagogy with computer-assisted language learning and social robotics.

Another research-driven project is *Early Language Development in the Digital Age*, or “e-LADDA”.6https://www.ntnu.edu/e-ladda/e-ladda. The project, which runs from 2019 to 2024, is a collaboration between academics, the non-academic public sectors and technology companies in the industry. It aims to investigate how digital tools affect language development and performance in young children, as well as to improve understanding of the technology itself and how it is used. The project’s primary goal is to provide a unified research methodology for studying how digital technologies affect early childhood language learning and to provide guidelines for policymakers, educators, practitioners and families on navigating, regulating and adapting to emerging digital environments.

## 4 Evaluation of RALL

As described above, robots have good potential for second language learning, both technically and in terms of application. So, how effective is robot-assisted language learning? Generally speaking, the effects of RALL have been positive with a medium average effect size, according to a meta-analysis in 2022 (Lee & Lee, 2022). This study has shown that language learning improvement has been achieved under RALL conditions, regardless of moderator variables (e.g., age group, target language, robots’ role, interaction type). In more detail, the evaluation of RALL can be divided into the following categories: (1) cognitive and affective learning gains for students. The former refers to the achievement of learning language skills. The latter refers to students’ motivation, confidence and social behaviours; (2) teaching strategies under RALL; (3) technological aspects of robots.

### 4.1 For language learners

Among the available RALL studies, vocabulary learning has taken up the largest proportion, followed by reading and speaking skills, with minimal research done on grammar learning (Van den Berghe et al., 2019). From the learners’ perspective, studies have shown that RALL has generated positive results in helping L2 learners to acquire reading (Hong et al., 2016) and grammar skills (Herberg et al., 2015; Kennedy et al., 2016). According to (Van den Berghe et al., 2019), there is a mixed picture in terms of vocabulary learning and speaking. As for affective gains, as discussed earlier, L2 learners tend to be highly motivated by the physical presence of and interaction with robots, especially in the short term. However, it is not clear how much a feeling of novelty has impacted the L2 learners’ performance. According to the extensive review (Randall, 2019), students’ classroom participation and self-confidence were improved under RALL. Additionally, contradictory results about RALL’s influence on L2 learners’ social behaviours are given. For example, L2 learners did not show any lexical and syntactic alignment when they spoke to an embodied robot or the virtual agent (Rosenthal-von der Pütten et al., 2016). However, L2 learners have shown different responses to the robot’s feedback: sadly, punishment feedback is shown to be more effective than reward feedback (de Haas & Conijn, 2020).

### 4.2 For language teachers

From the teachers’ perspective, language teaching and learning is a continuously interactive activity which requires collaboration as much as other spoken interactions (Holtgraves, 2013). Thus, it is worth finding out how to work with robots in the language classroom effectively. Olov’s review has shed some light on this aspect by reviewing teaching strategies and how they are combined with robots used in RALL (Engwall & Lopes, 2022). It has inspired creativity in language classrooms. Robots can take on different roles, either as teaching assistants, learning companions, or even as ‘little villains’ who deliberately make mistakes, depending on the complexity of tasks and freedom in human-robot interactions. A more specific review of RALL’s oral interaction, including 22 empirical studies from 2010 to 2020, shows that communicative language teaching (CLT) is most widely used in RALL, followed by teaching proficiency through reading and storytelling (TPRS) (Lin et al., 2022). It also details what actions teachers took in those experiments to collaborate with robots.

### 4.3 For technologies used in RALL

From the robot developers’ perspective, studies of RALL have flagged many aspects to consider, including robots’ form, voice, social roles and behaviours (Randall, 2019). For example, with the advantage of embodiment, the robot can also use encouraging gestures to enhance learning (Harinandansingh, 2022). In theory and practice, building a language-learning-assisted robot is achievable. Qilin, a Chinese vocabulary-learning robot, is an example (Nomoto et al., 2022). It utilised mature Application Programming Interfaces (API) like Google Cloud on a low-cost computer like Raspberry Pi. It was tailored for specific language-learning purposes in the experiment. Whilst using multimodal cues like lights and movement enhances the interactive effects, the choice of Qilin’s voice seems not liked by students. Plus, Qilin’s camera does not provide any practical use other than making the robot look more like a human.

Notably, while developers may well want human-robot interaction to be as natural as human interaction, this does not mean that every aspect of the robot has to be as human-like as possible. The design of robots’ behaviours and affordances is not only a technical but also an ethical issue (Hildt, 2021; Huang & Moore, 2022). For example, people could react negatively to a robot’s deceptive praise (Ham & Midden, 2014).

### 4.4 Session summary

The above assessment of RALL is only a glimpse into the whole picture. Most of the RALL at this stage has focused on children and adolescent second language learners, with less research on adult second language acquisition. The different tasks were used in different studies, with different experimental designs, sizes of participants and lengths of human-computer interaction, or even lack of control conditions. All of these add to the difficulty of cross-sectional comparisons and come to cogent conclusions.

## 5 Discussion and opportunities

Born around the mid-2000s, RALL is still a very young field. This means that most of the RALL studies are exploratory. Although the potential of RALL is considerable, there is still a long way to go before the widespread use of robot-assisted teaching within second-language classrooms (Shadiev & Yang, 2020). RALL is an interdisciplinary field that requires the collaboration of language teachers, language acquisition theorists, psychologists, educators, and robotics and communication technologists. There is still a lot of work that needs to be done.

### 5.1 Technological challenges

In theory, RALL has essential advantages that other technology-based language teaching tools do not have. It is important to note that these advantages are currently underdeveloped in reality (Van den Berghe et al., 2019). One of the reasons is that, compared to CALL, RALL has to face a more dynamic interactive environment. This set higher demands for coherence and robustness of language-learning-assisted robots. From the designer’s perspective, misalignment would cause conflicting perceptions, which could lead to decreased motivation to interact with a robot (Meah & Moore, 2014). As for the state-of-art artificial intelligence, visual recognition system, automatic speech recognition system and dialogue system are not yet sufficient to allow the robot to automatically and fluently talk to any second language learner, partially because L2 speakers’ speeches are more unpredictable and more difficult to recognise. Also, a significant advantage of the robot is the ability to interact with the teaching environment and students using an embodiment. This advantage is not fully utilised. It is hoped that developers will soon break down the limitations of robotics (e.g. stability in movement, overheating issues) and allow the advantages of the robot to be better utilised.

In addition to these matters, the technical challenges faced by RALL require consideration of the specificities of language teaching and learning. Evaluation criteria in academia or industry may not be the most appropriate standard for language learning. Take the Automatic Assessment of Pronunciation (AAP) in L2 for example. The principle of robot judgement is to compare the sound produced by L2 learners with the sound that the system considers correct. This judgement started from the phoneme-based comparison in the early days (e.g., distinguishing between voiced stops and voiced fricatives (Weigelt et al., 1989)), with a shifted focus on fluency at the sentence level (Bernstein et al., 2011; Kim et al., 1997), and then has developed further by taking into count the influence of social factors (e.g., age, gender) (Strand, 1999). In recent years, the importance of measuring genuine listener intelligibility has attracted more attention. Instead of native-like accuracy, clear speech has become a more realistic goal for intelligibility assessment (O’Brien et al., 2018) but has yet to further develop or commercialise.

Thus, adopting automatic judgement in language learning requires careful consideration. It might be worth considering if teaching aims to get students to pronounce exactly like L1 speakers. But the question is, what are these supposedly correct L1 speakers’ data that are used to train the model? Why should L2 speakers’ pronunciation be identical to L1 speakers’? How good is L2 users’ pronunciation good enough? How important is the pronunciation of words and sentences in oral interactions? What is the impact of emphasising correct pronunciation on the development of students’ oral or general language skills? If these questions cannot be answered, it may be unwise to promote technology-based pronunciation assessment, including RALL. Thus, on the one hand, it is essential to adopt the human-centred approach to develop more adaptive assessment tools to suit the diverse needs of second language learners. On the other hand, product developers are expected to enhance the transparency and explainability of the technology. This will make it easier for users to (1) choose the appropriate context of use and (2) judge how to interpret the results obtained.

Another aspect to consider is the relationship between robots and humans. Given a robot is not a one-off disposable product in language classrooms, can the robot maintain a long-term, healthy relationship with the student? Also, the language learning effect could be variable. Can a robot adapt its behaviour (e.g., feedback) to the student’s individual characteristics and difficulties? Additionally, the interaction between RALL and students and teachers is bound to generate a lot of data. Data collection, storage and sharing is also an ethical matter worth considering.

### 5.2 Opportunities for L2 teachers

As mentioned earlier, the novelty that the robot brings to the students is likely to wear off after a few interactions. Therefore, if L2 teachers are interested in trying RALL, they must consider introducing the robot as a new teaching tool to their students to reduce the novelty effect. A more pragmatic approach for language teachers is to be grounded in reality, identify the real-world problems that need to be addressed, understand the shortcomings of existing products, and then see what can be done and how far it can go within the confines of the syllabus based on the current level of technology.

Would robots replace language teachers on various fronts? Teaching is not a simple process of telling students what to do but a multi-linked activity. The teacher’s function involves selecting, organising, and presenting materials, monitoring the effectiveness of student learning, giving feedback, and adjusting one’s teaching to changes in the external learning environment and the needs of students. An experienced teacher can take into account all aspects and tailor the teaching to the needs of the students and the local context. Should or could a language-learning-assisted robot be as skilful and all-encompassing as that? Furthermore, interaction is a complex two-way collaborative process, and oral interaction is the challenging part of human-computer interaction (Moore, 2016). For language teaching, the interaction between teachers and students contains many other elements besides the transmission of knowledge and information, and further research on its complexity is needed. After all, robots, like other technology-based teaching tools (e.g., PowerPoint), can serve certain aspects of teaching and learning. The key is not to use it or not, but to use it in what situations and how.

While waiting for the technology to mature, teachers can increase their knowledge of RALL and familiarise themselves with this burgeoning technology. This is not only in preparation for future classroom use but also to facilitate participation in the development of RALL. This requires AI’s cognitive mechanics and interactive capabilities. That makes artificial social interaction ‘one of the most formidable challenges in artificial intelligence and robotics’ (Belpaeme et al., 2018). Teachers have an important role to play in the development of RALL. Here are some potential directions to explore.

– **RALL Teacher Training**: One example is to consider teacher training related to RALL, from theory (Mishra & Koehler, 2006) to practice (Li & Tseng, 2022).
– **Non-language Aspects in L2 Acquisition**: Another example is to expand the application of RALL from language teaching to cultural teaching to help students understand the social etiquette and customs of the target language country (Wallace, 2020).
– **Work Around the Limits:** Consider innovative ways to work with the limits of robots, like having 2-to-1 group interaction instead of 1-2-1 interaction between robots and students (Engwall et al., 2021; Khalifa et al., 2018).

## 6 Conclusions

With the development of robotics and communication technology, using robots in language teaching has become possible. The emerging field of RALL has aroused much interest and enthusiasm. This article introduces the possibility and necessity of the birth of RALL from the perspective of the development of robotics, provides a brief review of RALL research in terms of both technology and applications, and points out existing problems and opportunities for development.

The general language learning environment is changing, and the learning tools are evolving. Such changes have brought in possibilities. RALL has been at the stage of exploring these possibilities. The current state of the art is that there are no all-round language-teaching robots proficient in listening, speaking, reading and writing. Although limited in practice, RALL has offered a wide array of potential ways to reshape language teaching and learning, especially for a broad yet under-explored area like Chinese as a foreign language. Chinese language teachers can refine their language teaching and learning needs, collaborate with robot developers to design different modes of RALL interaction, and conduct empirical experiments and comparative studies. In the experimental design, care should be taken to meet empirical standards (e.g., the sample size and use of control groups) in order to provide more conclusive evidence of these technologies.

Drawing on the development of human-computer interaction (HCI), it is believed that the interactivity of human-robot interaction (HRI) will improve, and the user group of RALL will be enlarged from robotics experts and amateurs to an extensive range of ordinary people. It requires tightly integral endeavours to solve technical challenges and change educational practices (Belpaeme et al., 2018). The challenge is for the market, language teaching institutions, research institutions, and government if they can work together. In that case, it will help deepen the understanding of the problem from multiple perspectives, find the middle ground between what is feasible and what is needed, identify the challenges and outline the future of RALL.
